# Subcutaneous Infection Model Facilitates Treatment Assessment of Secondary Alveolar Echinococcosis in Mice

**DOI:** 10.1371/journal.pntd.0002235

**Published:** 2013-05-23

**Authors:** Tatiana Küster, Corina Hermann, Andrew Hemphill, Bruno Gottstein, Markus Spiliotis

**Affiliations:** Institute of Parasitology, Vetsuisse Faculty, University of Berne, Berne, Switzerland; University of Nottingham, United Kingdom

## Abstract

Alveolar echinococcosis (AE) in humans is a parasitic disease characterized by severe damage to the liver and occasionally other organs. AE is caused by infection with the metacestode (larval) stage of the fox tapeworm *Echinococcus multilocularis*, usually infecting small rodents as natural intermediate hosts. Conventionally, human AE is chemotherapeutically treated with mebendazole or albendazole. There is, however still the need for improved chemotherapeutical options. Primary *in vivo* studies on drugs of interest are commonly performed in small laboratory animals such as mice and Mongolian jirds, and in most cases, a secondary infection model is used, whereby *E. multilocularis* metacestodes are directly injected into the peritoneal cavity or into the liver. Disadvantages of this methodological approach include risk of injury to organs during the inoculation and, most notably, a limitation in the macroscopic (visible) assessment of treatment efficacy. Thus, in order to monitor the efficacy of chemotherapeutical treatment, animals have to be euthanized and the parasite tissue dissected. In the present study, mice were infected with *E. multilocularis* metacestodes through the subcutaneous route and were then subjected to chemotherapy employing albendazole. Serological responses to infection were comparatively assessed in mice infected by the conventional intraperitoneal route. We demonstrate that the subcutaneous infection model for secondary AE facilitates the assessment of the progress of infection and drug treatment in the live animal.

## Author Summary

Alveolar echinococcosis is a disease which affects humans and inflicts severe damage to the liver and other organs. It is caused by a parasite whose definitive host is the fox. Despite being a relatively rare disease, an increasing number of new cases has been reported in central and eastern European countries more recently. The current therapy in human AE patients consists of benzimidazoles. The treatment has to be taken on a daily basis for very long periods of time, or even lifelong. New options are currently being searched for, mainly based on compounds that show efficacy in experimental animal infection models. The infection is commonly done by injecting parasites directly into the peritoneal cavity of the animals, with risk of damage to the surrounding organs. The efficacy of applied treatments can only be evaluated at the end of the studies by dissection of the animals. In this study we show that the subcutaneous infection model can be applied for drug treatment trials and enables the direct monitoring of treatment effects during the entire study period.

## Introduction

*Echinococcus multilocularis*, known as the fox tapeworm, is a parasite of the Taeniidae family, whose metacestode (larval) stage causes the zoonotic disease “alveolar echinococcosis (AE)” in the intermediate hosts including humans. [1]. The disease displays a clinical manifestation similar to that of a slow-growing malignant tumor of the liver. The continuously proliferating parasite progressively invades the liver parenchyma, bile ducts and blood vessels, which results in symptoms related to biliary obstruction, portal hypertension and others. Secondary infection upon dissemination of metacestode structures via the blood or lymphatic system is most commonly found in adjacent organs such as the lungs, but also in the brain [2] and other more remote sites.

The treatment options for AE are surgery and/or chemotherapy. The feasibility of surgery depends on the number, location and extension/size of the metacestode lesion. In addition, care must be taken to remove the entire parasite mass, otherwise recurrences might occur, and there is the risk of metastasis formation by accidental dissemination of small daughter vesicles, cells or cell-conglomerates. Surgery (radical or palliative) is always accompanied by pre- and post-operative chemotherapy. In cases where surgery is not possible, chemotherapy remains the only option. For chemotherapeutical treatment of AE, the only two drugs licensed to date are the benzimidazole carbamate derivatives albendazole and mebendazole. Both exhibit a relatively good clinical efficacy but side effects may occur, and for most cases the drugs do not act parasitocidal [3], [4].

In order to identify better drugs for the treatment of AE, several compounds have been investigated in the past, either employing *in vitro* cultured parasites and/or *in vivo* rodent models [3], [5]. The secondary murine AE model is currently used for experimental treatment trials [6]. In this model, the parasite metacestodes are injected into the peritoneal cavity of a mouse or a Mongolian jird (*Meriones unguiculatus*), where the parasites proliferate and develop tumour-like features such as progressive growth and invasion of neighbouring tissues. Considerable organ damage might be inflicted by injection, and parasite growth can only be assessed by the dissection of the animals at the end of the study.

In the frame of the present study, we propose that subcutaneous injection of the parasite represents a viable alternative. This route represents, in comparison to the intraperitoneal route, a reduced risk of organ damage and of secondary infection. Subcutaneous models have been used in animal models for cancer studies since 1946, and offer the advantage that tumors are readily visible from the outside, thus facilitating the direct visual monitoring of cancer progression upon treatment [7]. Subcutaneously injected metacestodes have been recently employed to prove the efficacy of radiotherapy [8]. The aim of the present study was to verify whether the subcutaneous infection model for AE was also applicable to monitor and assess the effects of chemotherapy.

## Materials and Methods

### Media and biochemicals

If not stated otherwise, all culture media and reagents were purchased from Gibco-BRL (Zürich, Switzerland) and biochemical reagents were from Sigma (St. Louis, Mo, USA). Albendazole was purchased from Sigma (St. Louis, Mo, USA).

### *In vitro* culture of *E. multilocularis* metacestodes

Culture of *E. multilocularis* (isolate H95) was carried out as previously described [9], [10]. In short, metacestodes dissected from experimentally infected Balb/c mice were pressed through an autoclaved metal tea strainer. The metacestodes were incubated in antibiotic solutions: 20 µg/mL Levoflaxin (Aventis, Meyrin, Switzerland) and 20 µg/mL Ciprofloxacin (Bayer, Zürich, Switzerland), in PBS overnight. The sedimented material was washed several times with 1× PBS and 1 mL was added to a cell-culture flask containing 5×10^6^ rat hepatoma (ATCC: CRL-1600) cells/50 mL cultivation-medium (DMEM, 10% FCS, 100 U/mL Penicillin G, 100 µg/mL Streptomycin sulphate). These co-cultures were incubated at 37°C, 5% CO_2_, with medium changes once a week. Splitting of cultures was carried out when exceeding 15 mL total metacestode volume. Metacestodes were used for experimental procedures when they reached diameters of ∼4 mm.

### Comparative studies on the efficacy of albendazole treatment in Balb/c mice experimentally infected through the subcutaneous or intraperitoneal route

Twenty-four female Balb/c mice (age 9 weeks; body weight 20–25 g) were housed in a temperature-controlled light cycle room with food and water *ad libitum*. Experiments were carried out according to the Swiss Animal Welfare regulations, and experiments were approved by the Board for Supervison of Animal Research of the Canton of Bern (license No. BE103/11). 10 mice were infected by intraperitoneal (ip) injection of metacestodes, and another 10 mice were infected subcutaneously (sc), each with 100 µl of homogenized *in vitro* cultivated metacestode material (isolate H95). 4 control animals remained uninfected. After 6 weeks, the infected animals were allocated into 4 experimental groups with 5 mice each: (i) sc infection and albendazole treatment (scABZ); (ii) sc infection and mock treatment (sc-untreated); (iii) ip infection and albendazole treatment (ipABZ); (iv) ip infection and mock treatment (ip-untreated). Additionally, 2 of the uninfected animals were treated with albendazole and 2 were mock-treated (controls). Drug treatments were carried out by daily oral application of albendazole (200 mg/kg) suspended in 100 µl of a 1∶1 mixture of honey (M-Budget honey, Migros Switzerland) and 1% CMC (carboxymethyl cellulose sodium salt) as described earlier [11]. Mock treatments were performed by oral application of 100 µl honey/CMC. The animals were photographed and weighted periodically throughout the treatment.

At the end of the study the animals were euthanized, necroscopy was performed, the entire parasite tissue was recovered and the parasite weight was determined. Several samples of the recovered material were processed for TEM. Parasite weights were analyzed by boxplots, and outliers were identified by the ESD method (extreme studentized deviate) with a significance level of 0.05 (two-sided). Only one outlier was identified and discarded in the subcutaneously infected and treated group, which showed *P*>0.05 in relation to the other values within the group. The remaining data were submitted to a two-tailed distributed student t-test, with two-sample equal variance between the untreated groups and the treatment groups. The uninfected control animals were not included in the calculations.

### Serology

Blood was taken at the time of euthanasia by heart puncture and serum was obtained after centrifugation. The humoral immune response was assessed by ELISA, detecting specific antibodies against *E. multilocularis* antigens such as Em18 [12], Em2 [13] and vesicular fluid [14]. Vesicular fluid was obtained from *in vitro* cultured *E. multilocularis* metacestodes by gently breaking them with a pipette, and subsequent centrifugation at 600× *g* for 5 min at RT to separate vesicle fluid from vesicle walls. The supernatant (containing vesicle fluid) was collected, sterile filtered (0.2 µm), and stored at -80°C. Em2(G11)-antigen was obtained by affinity chromatography as previously described [15]. Recombinant Em18 was expressed and purified as described [16].

For ELISA, 96 well plates were coated overnight at 4°C with vesicular fluid (1 µg protein/mL), or Em2(G11)-antigen (2.8 µg carbohydrate/mL in a dilution of 1∶200) and recombinant Em18 (0.4 µg protein/mL) antigens diluted in coating buffer (0.05 M NaHCO_3_–Na_2_CO_3_, pH 9.6). The plates were then washed 3× with washing buffer (1.5 mM KH_2_PO_4_, 10 mM Na_2_HPO_4_, 150 mM NaCl, 2.5 mM KCl, pH 7.4), blocked for 30 min at 37°C with 100 µl per well of dilution buffer (washing buffer supplemented with 1% horse serum). Serum samples were diluted 1∶100 in dilution buffer and incubated in microplates for 60 min at 37°C. After extensive washing, the plates were incubated for 30 min at 37°C with goat anti-mouse IgG alkaline phosphatase (AP) conjugate (Promega, Madison, WI, USA) at a dilution of 1∶1000. Following three washes, the wells were incubated with 100 µl of AP substrate (1 mg/mL *p*-nitrophenyl–phosphate–disodium in 1 M di-ethanolamine containing 0.1 mM MgCl_2_, pH 9.8) for 30 minutes, and then the reaction was stopped by the addition of 30 µl 3 M NaOH. The absorbance at 405 nm was measured at room temperature in a tunable microplate reader (Dynatech, Embrach, Switzerland). Conjugate controls, blanks and mouse positive and negative controls were performed in parallel. For each sample duplicate measures were performed. The data was submitted to a 2-tailed, homoscedastic Student *t* test, with 2-sample equal variance.

### Transmission electron microscopy (TEM)

To visualize the structural alterations in metacestodes imposed by the albendazole treatment in both models, the parasites harvested at the time point of euthanasia were processed for transmission electronic microscopy (TEM). Briefly, metacestodes were fixed in 2.5% glutaraldehyde in 100 mM sodium cacodylate buffer (pH 7.2) for 2 h at room temperature, followed by postfixation in 2% OsO_4_ in 100 mM sodium cacodylate buffer (pH 7.2) for 2 h at room temperature. The fixed specimens were washed in distilled water, and were treated with 1% uranyl acetate for 30 min, dehydrated through a graded series of ethanol (30-50-70-90-3×100%) and were finally embedded in epoxy resin (Epon 812) with three changes of resin during two days. Resin polymerization was carried out at 65°C overnight. Sections of 80–90 nm thickness were cut on a Reichert and Jung ultra-microtome, loaded onto 300-mesh copper grids (Plano GmbH, Marburg, Germany), and stained with uranyl acetate and lead citrate as previously described [17]. Specimens were viewed on a Phillips EM400 transmission electron microscope operating at 80 kV.

## Results and Discussion

### Parasite burden of intraperitoneal (ip) and subcutaneous (sc) infection models

**Figure 1:**
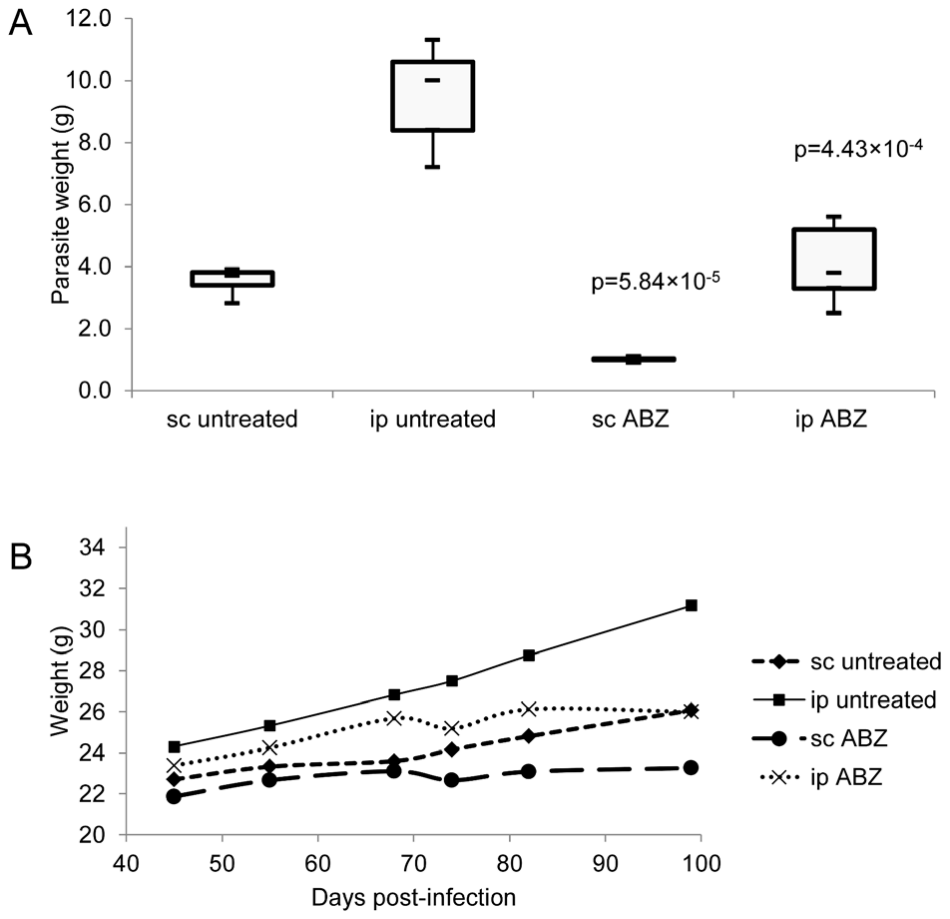
Parasite burden and weight control. **A**) Parasite burden of intraperitoneally (ip) and subcutaneously (sc) infected animal models. Albendazole treatment was initiated at 6 weeks post-infection. Each treatment group comprised 5 infected animals. scABZ and ipABZ groups received albendazole in honey/CMC, sc-untreated and ip-untreated groups received honey/CMC without compound. The *P* values indicate the scores obtained by student's t-test in comparison with the respective untreated groups. **B**) Weight control of mice throughout the study. Representation of average weight of different experimental groups at defined timepoints during the study. Treatments were initiated at day 42 p.i. The non-infected controls are not shown. The groups were divided into route of infection (subcutaneous = sc; intraperitoneal = ip), and in albendazole-treated (scABZ; ipABZ) or non-treated (sc-untreated; ip-untreated).

The parasite weights recovered from ip and sc infected Balb/c mice, treated or not with albendazole, are depicted in Fig. 1A.

The sc-untreated group yielded 63% less parasite material compared to the ip-untreated group. This difference in the progress of parasite development might be due to the more restricted physical space for growth provided in the sc infection model, or due to difference in the immune responses triggered by the presence of the parasites at the two different sites. This could also be observed throughout the treatment, reflected in the weights of the individual mice of different groups (Fig. 1B). As the weight of the animals can be correlated to parasite growth, the weight progression of the animals can also be used as a parameter for monitoring the effects of chemotherapy. This is confirmed by the steady weight increase of the untreated groups throughout the experiment, while the average weight of both treated groups reached a plateau around 70 days post-infection, or 30 days after the beginning of treatment, due to the effects of chemotherapy on parasite growth. Thus, the weight of the mice could be correlated to the growth of the parasite (Fig. 1B).

Daily oral application of albendazole yielded the expected results, namely a significantly decreased parasite weight [6], [18], [19]. The ipABZ and scABZ groups were compared to their respective ip-untreated and sc-untreated control groups. A decrease in the amount of harvested parasite material of 73% was observed in the sc infected mice (*P* = 5.84×10^−5^), while for the ip infected mice (*P* = 4.43×10^−4^) a decrease of 62% was observed.

**Figure 2:**
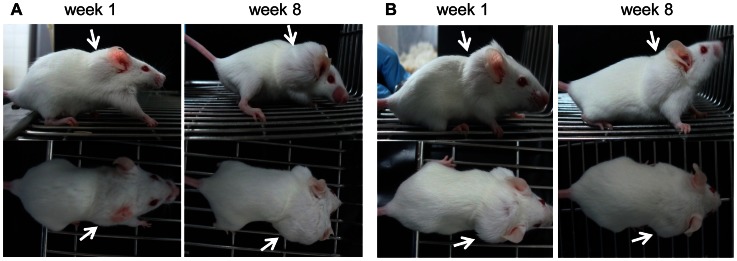
Visible monitoring of mice during treatment. The animals were photographed at week 1, and week 8 of treatment. The arrows indicate the site of subcutaneous infection. Subcutaneously infected animals are shown in panels A (untreated), and B (treated with albendazole). One example of each group is shown. Note that the parasite burden in B does not increase upon treatment. This is not visible in intraperitoneally infected mice, but detectable in the subcutaneous model despite the considerably impaired parasite growth due to albendazole treatment (not shown).

Besides the decreased risk of injury presented by the sc injection of metacestodes, the sc model also enables an easy monitoring of infection and treatment. This is shown in Figure 2. In the sc infected mice, the initial growth and subsequent inhibition of parasite growth upon treatment are both visible by eye (Figure 2B), but this does not hold true for the ip infected mice, where a visible reduction in parasite mass would only be evident upon even more prolonged treatment (unpublished observations). These results are consistent with the animal's progression of weight, as mentioned above. Thus, the sc infection model offers the additional advantage of enabling the researcher to detect potential treatment efficacy within a shorter time span compared to the ip infection model. Moreover, for experiments covering a longer time span, no mobility impairment was observed in the sc infected mice, in contrast to ip infected animals, where increased parasite mass can lead to impaired mobility. This is due to the location of the infection (ventral opposed to dorsal) and the virtually unrestricted parasite growth in the peritoneal cavity of infected animals.

Although no extensive studies were performed on potential metastasis of the parasite for both infection models, invasion of adjacent tissues was not observed in sc infected mice. This renders the sc infection model unsuitable for studies on metastasis formation. On the other hand, the sc infection model would permit injection of different *E. multilocularis* isolates into the same mouse at multiple sites.

### Structural damage of the parasite caused by drug treatment

**Figure 3:**
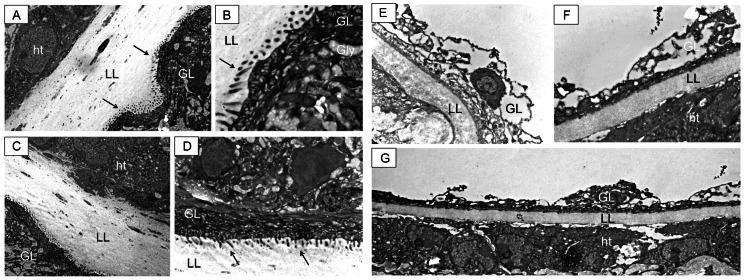
TEM of metacestodes recovered from untreated and subcutaneously infected treated mice. Metacestodes recovered from intraperitoneally (A and B) and subcutaneously (C and D) infected mice exhibit a well-defined laminated layer (LL), which is in close physical contact with adjacent host tissue (ht). The tegument, with microtriches (arrows) protruding well into the LL, and the germinal layer (GL) exhibiting undifferentiated cells and glycogen storage cells, are clearly visible. Metacestodes from animals subcutaneously infected and treated orally with albendazole show a reduced and less defined laminated layer (LL), which maintains contact with host tissue components. This is clearly visible in G. Other alterations can be seen in E and F, such as the loss of integrity of the germinal layer (GL) and the absence of microtriches (for comparison refer to A–D). Similar structural damage was observed on the material collected from intraperitoneally infected animals treated with albendazole (results not shown).

TEM was employed to visualize and compare the effects of albendazole treatment on ip and sc infected mice as shown in Figure 3.

Untreated *E. multilocularis* metacestodes exhibited the typical features described previously [20]. The most outer acellular and carbohydrate-rich laminated layer surrounding the entire parasite is clearly visible and in close contact with the host tissue. (Fig. 3 A–D). The live parasite tissue is localized just adjacent to the inner surface of the laminated layer, with the tegument forming microtriches that protrude into the laminated layer. The germinal layer is constituted by a relatively densely packed tissue containing muscle cells, connective tissue, undifferentiated cells, and mostly fully-loaded glycogen storage cells. TEM micrographs taken of the scABZ group show severe drug-induced alterations in the structural integrity of the germinal layer and a complete loss of microtriches (Fig. 3 E–G). These alterations are similar to those described earlier in metacestodes obtained from ip infected Balb/c mice [6]. In addition, the laminated layer seems to be reduced in width upon treatment, probably due to the fact that the parasite tissue forming the laminated layer has been severely metabolically impaired during treatment and synthesis as well as secretion of laminated layer components was halted (Fig. 3). Similar damage was observed in the ipABZ group (results not shown).

### Serological comparison of sc and ip infected mice

**Figure 4:**
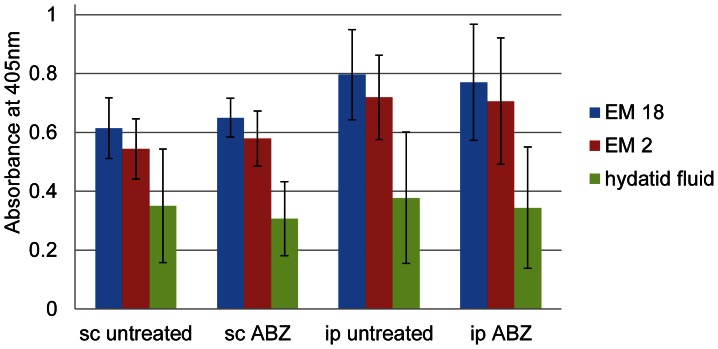
Serological analysis of infected mice 14 weeks post-infection. Antibody responses of subcutaneously (sc) and intraperitoneally (ip) infected mice, either treated with albendazole (ABZ) or not (untreated) were measured by ELISA employing recombinant Em18 (EM18), Em2(G11)-antigen (EM2) or hydatid fluid. Error bars indicate standard deviations. There is no difference in the responses of treated versus respective untreated animals, and no significant difference can be seen between the two infection models.

In order to characterize the humoral immune responses of the mice in the different infection models, blood samples were taken at the time point of euthanasia and serum was obtained. ELISA detected antibodies against Em18 [12], Em2 [13] and vesicle fluid [14]. The results in Figure 4 show that the ip-untreated or sc-untreated groups exhibit a largely identical antibody reactivity pattern, even though the antibody levels are slightly lower in sc infected mice. They showed no significant difference in the humoral immune response of both groups (*P* = 0.08 for Em18; *P* = 0.04 for Em2 and *P* = 0.85 for vesicle fluid). The same was observed for the scABZ and ipABZ treated groups (*P* = 0.23 for Em18; *P* = 0.23 for Em2 and *P* = 0.74 for vesicle fluid). Thus, in both infection models treatments with albendazole did not seem to affect the specific antibody synthesis.

### Conclusion

Using treatment with albendazole, we have shown that the sc infection model is suitable for investigating the effects of chemotherapeutically interesting compounds against secondary murine AE. This model has the advantage over the conventional ip model of facilitating visual monitoring throughout the course of treatment. It remains to be investigated whether it can be used to assess the effect of other drugs apart from albendazole, thus representing a general model for research concerning chemotherapy.
